# Intestinal Epithelial Cell Regulation of Adaptive Immune Dysfunction in Human Type 1 Diabetes

**DOI:** 10.3389/fimmu.2016.00679

**Published:** 2017-01-10

**Authors:** Christina L. Graves, Jian Li, Melissa LaPato, Melanie R. Shapiro, Sarah C. Glover, Mark A. Wallet, Shannon M. Wallet

**Affiliations:** ^1^Department of Oral Biology, College of Dentistry, University of Florida Health Science Center, Gainesville, FL, USA; ^2^Department of Gastroenterology, Hepatology, and Nutrition, College of Medicine, University of Florida Health Science Center, Gainesville, FL, USA; ^3^Department of Pathology, Immunology, and Laboratory Medicine, College of Medicine, University of Florida Health Science Center, Gainesville, FL, USA

**Keywords:** type 1 diabetes, mucosal immunity, intestinal epithelium, primary IEC, adaptive immunity, T cell proliferation, T cell polarization

## Abstract

Environmental factors contribute to the initiation, progression, and maintenance of type 1 diabetes (T1D), although a single environmental trigger for disease has not been identified. Studies have documented the contribution of immunity within the gastrointestinal tract (GI) to the expression of autoimmunity at distal sites. Intestinal epithelial cells (IECs) regulate local and systemic immunologic homeostasis through physical and biochemical interactions with innate and adaptive immune populations. We hypothesize that a loss in the tolerance-inducing nature of the GI tract occurs within T1D and is due to altered IECs’ innate immune function. As a first step in addressing this hypothesis, we contrasted the global immune microenvironment within the GI tract of individuals with T1D as well as evaluated the IEC-specific effects on adaptive immune cell phenotypes. The soluble and cellular immune microenvironment within the duodenum, the soluble mediator profile of primary IECs derived from the same duodenal tissues, and the effect of the primary IECs’ soluble mediator profile on T-cell expansion and polarization were evaluated. Higher levels of IL-17C and beta-defensin 2 (BD-2) mRNA in the T1D-duodenum were observed. Higher frequencies of type 1 innate lymphoid cells (ILC1) and CD8+CXCR3+ T-cells (Tc1) were also observed in T1D-duodenal tissues, concomitant with lower frequencies of type 3 ILC (ILC3) and CD8+CCR6+ T-cells (Tc17). Higher levels of proinflammatory mediators (IL-17C and BD-2) in the absence of similar changes in mediators associated with homeostasis (interleukin 10 and thymic stromal lymphopoietin) were also observed in T1D-derived primary IEC cultures. T1D-derived IEC culture supernatants induced more robust CD8+ T-cell proliferation along with enhanced polarization of Tc1 populations, at the expense of Tc17 polarization, as well as the expansion of CXCR3+CCR6+/− Tregs, indicative of a Th1-like and less regulatory phenotype. These data demonstrate a proinflammatory microenvironment of the T1D-duodenum, whereby IECs have the potential to contribute to the expansion and polarization of innate and adaptive immune cells. Although these data do not discern whether these observations are not simply a consequence of T1D, the data indicate that the T1D-GI tract has the capacity to foster a permissive environment under which autoreactive T-cells could be expanded and polarized.

## Introduction

Type 1 diabetes (T1D) is a polygenic disease resulting from the triad of β-cell fragility, failure to regulate innate immunity, and failure to regulate adaptive immunity (1–3). To complicate matters, it is appreciated that environmental factors contribute to the initiation and progression of T1D, yet a single environmental trigger has not been identified (4). Thus, we propose that rather than solely the identity of the trigger, the nature of the host responses to environmental triggers is also contributing to the etiology of T1D. Integral to the sensing of the environment is the gastrointestinal tract (GI), which is the largest environmental interface in the human body (5). In health, the GI tract acts not only as a gatekeeper allowing absorption of nutrients and limiting passage of dietary, bacterial, and viral antigens but also contributes to the maintenance of immune tolerance (6). Previous studies have demonstrated that diet, changes in commensal microbiota, and alterations in intestinal permeability can all modulate autoimmune diabetes in animal models (7, 8). In addition, studies in humans have found associations between subclinical intestinal immune activation with T1D, as measured by local cytokine expression and frequency of both regulatory (Tregs) and inflammatory (Th17 cells) immune cells (7, 8). While these data implicate alterations in GI-sensing of the environment in individuals with T1D, the associated mechanisms leading to the observed immune microenvironment are yet to be described.

For instance, innate lymphoid cells (ILC), a recently described class of innate immune cells critical in maintenance and regulation of mucosal homeostasis through modulation of both innate and adaptive immunity, have not been evaluated in T1D (9–12). Similarly, it has been demonstrated that intestinal epithelial cells (IECs) upregulate innate immune functions at the expense of nutrient absorption indicating a dichotomous state where under conditions of immune dysregulation IEC may function primarily as innate immune effector cells. Here, IECs can biochemically regulate innate and adaptive immune cell populations (13, 14), yet IEC-innate immune function has also not been evaluated in T1D. This is in part due to the inaccessibility of sufficient and appropriate GI tissues from individuals with and without T1D. In collaboration with the Network for Pancreatic Organ Donors (nPOD), we have established methods to not only isolate and characterize immune cell populations within human duodenal tissues but to also establish primary IEC cultures from the same tissues (15). Thus, in order expand on the existing knowledge associated with the GI-immune microenvironment observed in T1D, we evaluated the soluble mediator milieu as well as the frequency and phenotype of ILC, T helper cells (CD4+), and cytotoxic T-cells (CD8+) within duodenal tissues in a cohort of organ donors with and without T1D. In order to begin to delineate mechanisms contributing to the observed GI-immune microenvironment, the resting IEC-innate immune profile of primary IECs derived from the same duodenal tissues was evaluated, and the effect of the innate immune profile on adaptive immune cell expansion and polarization was characterized.

## Materials and Methods

### Participant Cohort and Study Design

Fresh duodenal tissue was obtained from the nPOD (Gainesville, FL, USA). All experiments were performed according to the guidelines of the University of Florida Institutional Review Board, with written informed consent from all subjects. All subjects gave written informed consent in accordance with the Declaration of Helsinki. The protocol was approved by University of Florida Institutional Review Board. Included in the current study were 17 T1D and 13 non-T1D cases (Table S1 in Supplementary Material). When the proximal duodenum is included with pancreases donations, the duodenal tissue is harvested following the isolation of pancreatic tissues, pancreatic lymph nodes, spleen, and non-pancreatic lymph nodes. Specifically, the duodenum is opened and the mucosa cleaned of mucous and bacterial content with a moistened gauze pad after which a section is placed in transport media and immediately processed as follows. While the all organs are harvested according to the same protocol in every case, the time from death to organ harvest and thus confounders such as ischemia are not available. For each case, 8.0 g of duodenal tissue was processed as follows: (1) 0.3 g of tissue was preserved for (1a) protein extraction and (1b) RNA extraction to evaluate the soluble mediator profile of the duodenum by ELISA and real-time PCR, respectively. (2) The remaining tissue was processed for (2a) intestinal immune cell isolation and (2b) intestinal crypt isolation for characterization of resident immune cell population by flow cytometry and establishment of primary IEC cultures, respectively (15). (3) Following the establishment of confluent primary IEC cultures (3a) culture supernatants and (3b) cellular mRNA were collected to evaluate the soluble mediator profile of the primary cultures by ELISA and real-time PCR, respectively. (4) In order to evaluate the effect of the T1D and non-T1D-derived IEC-soluble mediator profile on T cell proliferation and polarization, T cells were isolated from a single systemically healthy individual (not part of the experimental cohort) and activated in the presence of supernatants from resting IEC cultures described in step 3, after which the subsequent T cell proliferation and polarization was characterized by flow cytometry. Please note while all samples (*n* = 17 T1D and *n* = 13 non-T1D) underwent evaluation for experiments outlined step 1, due to timing of receipt, only a subset of these samples (*n* = 8 and *n* = 10) underwent evaluation for experiments outlined in steps 1–4 (Table 1; Table S1 in Supplementary Material).

**Table 1:** **Cohort demographics**.

Group 1	Type 1 diabetes (TID) (*n* = 17)	Non-T1D (*n* = 13)
Age (±SEM)	30.8 (±3.28)	23.9 (±5.02)
Duration (±SEM)	16.8 (±3.24)	n/a
Sex (M/F)	12/5	10/3
Group 2	T1D (*n* = 8)	Non-T1D (*n* = 10)
Age (±SEM)	34.3 (±5.77)	26.6 (±6.30)
Duration (±SEM)	19.13 (±6.40)	n/a
Sex (M/F)	6/2	7/3

*Cases in Group 1 underwent analysis to evaluate the soluble mediator profile of the duodenum by ELISA and real-time PCR. Cases in Group 2 are a subset of those in Group 1 that also were subjected to analysis for resident immune cell population by flow cytometry and establishment of primary intestinal epithelial cell (IEC) cultures and the effect of IEC-soluble mediator profile on T cell proliferation and polarization. Please note, there are no statistical differences in the age, duration, and/or sex distribution between groups and/or disease diagnosis*.

### Whole Tissue Protein and mRNA Extraction

A total of 0.3 g of duodenal tissue was minced and homogenized by bead beating in cell extraction buffer (Life Technologies, Carlsbad, CA, USA) prepared with a protease inhibitor cocktail (cOmplete, Roche, Basel, Switzerland) and PMSF protease inhibitor (Life Technologies). Protein samples were stored at −80°C until ELISA could be performed. An additional 0.3 g of duodenal tissue was minced and homogenized by bead beating in Buffer RLT (Qiagen, Hilden, Germany) containing β-mercaptoethanol (β-ME) and total RNA harvested using an RNeasy extraction kit (Qiagen, Hilden, Germany) and also stored at −80°C until quantitative PCR (RT-qPCR) could be performed.

### Intestinal Crypt and Immune Cell Isolation

Crypts and resident immune cells were liberated using a protocol adapted from Booth and O’Shea (15–17). Human duodenal tissues were prepared by removing the longitudinal muscle layer and washing with ice-cold Mg^2+^- and Ca^2+^-free Hank’s Balanced Salt Solution (HBSS) (Mediatech, Manassas, VA, USA) containing 100 U penicillin, 100 μg/mL streptomycin (Mediatech), 25 μg/mL gentamycin (MP Biomedicals, Solon, OH, USA), and 0.5 mM dithiothreitol (DTT) (Thermo Fisher Scientific). After which the tissue was cut to yield 2 cm^2^ pieces, suspended in 50 mL of HBSS wash solution, inverted vigorously 10 times, and the contents allowed to settle for 1 min. The supernatant was removed and the process repeated four times. After the fifth wash, the settled contents were removed, minced with a sterile surgical scalpel, and suspended in 50 mL of the HBSS wash solution. The resulting solution was passed over a 400 μm^2^ filter, and the flow-through was collected for resident immune cells analysis (data not shown). The remaining tissue was digested in 50 mL of a buffer containing 75 U/mL collagenase type XI (Sigma-Aldrich, St. Louis, MO, USA), 20 μg/mL dispase neutral protease II (Roche, Indianapolis, IN, USA), 0.5 mM DTT, and 1% v/v fetal bovine serum (FBS) (Thermo Fisher Scientific) in Dubelcco’s Modification of Eagles Medium with 4.5 g/L glucose and l-glutamine, without sodium pyruvate (DMEM) (Corning, Corning, NY, USA). The digestion buffer containing the tissue was then evenly divided, placed in a 37°C incubator, and allowed to shake at 180 RPM for 3 h. The resulting digestion mixture was again passed over a 400 μm^2^ filter, and the tissue fragments atop the filter were washed with 25 mL complete growth media {DMEM [8.5 g/L sodium pyruvate (Mediatech)], 2.5% v/v FBS, 0.25 U/mL insulin (Sigma-Aldrich), 100 U penicillin, 100 μg/mL streptomycin, 25 μg/mL gentamycin, 5 μg/mL transferrin (Sigma-Aldrich), and 10 ng/ml epidermal growth factor (Sigma-Aldrich) containing 2% w/v d-sorbitol (Sigma-Aldrich)}. The tissue debris remaining in the filter was discarded, and the flow-through containing proliferative crypt structures and liberated intestinal immune cells was centrifuged at 200 × *g* for 4 min. The resulting pellet of intestinal crypts was used for the establishment of IEC cultures as described below. The resulting supernatant was collected for resident immune cells, which were cryopreserved at 1 × 10^6^ cells/mL in cell freezing media (ScienCell Research Laboratories, Carlsbad, CA, USA) and stored in liquid nitrogen until flow cytometry could be performed.

### IEC Culture and Stimulation

Intestinal epithelial cell cultures were established from isolated crypts as previously described (15, 17) and maintained in 24-well collagen-coated culture dishes (Greiner Bio-One, Monroe, NC, USA) in 1 mL complete IEC (cIEC) media [DMEM, 5 g/L sodium pyruvate (Mediatech), 2.5% v/v FBS, 0.25 U/mL insulin (Sigma-Aldrich), 100 U penicillin, 100 µg/mL streptomycin, 25 µg/mL gentamicin, 5 µg/mL transferrin (Sigma-Aldrich), 10 ng/mL epidermal growth factor (Sigma-Aldrich)]. Cultures were left unstimulated for 24 h after which supernatants were collected and stored at −80°C. In addition, cellular total RNA was harvested using an RNeasy extraction kit (Qiagen, Hilden, Germany) and stored at −80°C until RT-qPCR could be performed.

### Soluble Mediator Analysis

Reverse transcription and RT-qPCR was performed according to *MIQE* guidelines (18). Synthesis of cDNA was performed using SuperScript^®^ Reverse Transcriptase (Life Technologies), whereby RT-qPCR was performed using primer sets (Table S2 in Supplementary Material), and SsoAdvanced™ SYBR Green Supermix was used according to manufacturer recommendations. Data were collected and analyzed using CFX Connect™ and CFX Manager™ (Bio-Rad Laboratories, Berkeley, CA, USA) according to the *ddCT* algorithm using 18S as a reference gene and are presented as fold change. ELISA technology (EMD Millipore, Billerica, MA, USA) was used according to manufacturer protocol to assess whole tissue and primary IEC production of interleukin 10 (IL-10). Data were analyzed using a standard curve, positive, and negative control. For whole tissue expression, all data were normalized to total protein, while media-only levels were subtracted from primary IEC expression, which was normalized to 18S RNA levels from each primary cell culture.

### Flow Cytometry

Cryopreserved intestinal immune cells were thawed from liquid nitrogen at 37°C, washed, and suspended in PBS prior to incubation with a fixable Live/Dead Yellow viability dye (Life Technologies) for 10 min at RT. Following Fc receptor blocking (Human TruStain FcX™, BioLegend), surface staining was performed in FACS buffer [PBS, 1% FBS, 4 mM EDTA, and antibiotics (penicillin, streptomycin, and amphotericin B)] interrogating expression of lineage markers (CD3, CD14, CD19, CD20), CD56, CD127, CD117, NKp44, CD45, CD3, αβ TCR, γδ TCR, CD4, CD8, CCR6, CXCR3 (Table S3 in Supplementary Material). Intracellular staining for FOXP3 and HELIOS was performed using FOXP3 Fix/Perm Buffer Set (BioLegend). All antibodies were used at manufacturer-recommended concentrations. Fluorescence minus one or isotype controls were used as indicated. Data were acquired using a BD LSR Fortessa (BD Biosciences, Franklin Lakes, NJ, USA) cytometer and analyzed using FlowJo data analysis software (FlowJo, LLC, Ashland, OR, USA). All data were normalized to 1,000,000 total cells collected in the lymphocyte gate and are presented as frequencies and total cell numbers.

### Proliferation Assays

Peripheral blood mononuclear cells (PBMC) were isolated from the buffy coat of a single healthy donor (not part of the experimental cohort) by centrifugation (400 × *g*, 30 min, no brake) on Ficoll-Paque Premium (GE Healthcare Life Sciences, Pittsburgh, PA, USA). Cells were washed twice with HBSS and plated at a density of 1 × 10^6^ cells/mL with either 50% cRMPI/50% cIEC, or 50% cRPMI/50% 24-h supernatants from T1D- or non-T1D-derived IEC cultures. T cells were activated with Dynabeads^®^ Human T-Activator CD3/CD28 (Life Technologies) at a 1:1 bead to cell ratio with IL-2 (100 U/mL) for 4 days. CellTrace™ Violet-cell proliferation kit (Life Technologies) was used according to manufacturer protocols to evaluate CD3+ proliferation. Proliferation index was defined as the average number of divisions of each T cell as calculated by 1/sum[*p_n_*/(2*^n^*)].

### Histopathological Assessment

Hematoxylin- and eosin-stained sections of duodenum from all cases were obtained from nPOD and blindly evaluated by an experienced histopathologist (Jian Li) according to Marsh–Oberhuber classification (19) (Table S4 in Supplementary Material). Additional 5 µm sectioned slides of duodenum from all cases were used to assess goblet cell frequency by Periodic-acid Schiff staining (Sigma-Aldrich, St. Louis, MO, USA) according to the manufacturer protocol. Additional 5-µm tissue sections of duodenum from all cases were used to assess expression of HLA class II. Specifically, sections were deparaffinized and rehydrated prior to antigen retrieval in a sodium citrate buffer (10 mM sodium citrate, 0.05% Tween-20, pH 6.0; 96.5°C, 20 min). Slides were rinsed with PBS and permeabilized in a blocking buffer [1% (w/v) BSA, 10% (v/v) donkey serum, 0.1% (v/v) Triton X-100 in PBS]. Primary antibodies were used to probe for HLA-DR (TAL.1B5, Dako, Carpinteria, CA, USA) (16 h, 4°C, 1:20 in blocking buffer). Following three washes in PBS containing 3% (w/v) BSA and 0.1% (v/v) Triton X-100, tissue sections were probed with donkey anti-mouse AlexaFluor^®^ 488 (Life Technologies) (3 h RT, 1:400 in PBS). Following three washes in PBS, sections were mounted with VECTASHIELD^®^ HardSet Antifade (Vector Laboratories, Burlingame, CA, USA). Images were acquired with an EVOS^®^ FL (Life Technologies) and analyzed and quantified using ImageJ software (20). Data are presented as the mean fluorescence intensity difference of the region of interest and background. Exposure time and brightness and contrast adjustments were applied identically across all groups. A rainbow LUT was applied for visualization.

### Statistical Analyses

Data are expressed as mean ± SEM unless otherwise noted. Differences between groups were analyzed by the one-tailed Mann–Whitney *t* test using GraphPad Prism 6 software (GraphPad Software, San Diego, CA, USA). Differences in fold change of expression were analyzed by Wilcoxon matched-pairs signed rank test. Results were considered statistically significant at *p* ≤ 0.05.

## Results

### T1D-Associated Intestinal Innate Immune Activation

Previous studies using small intestinal biopsy samples from relatively small cohorts of children with T1D have suggested intestinal immune activation in some individuals with T1D (7, 8). In order to determine if this altered intestinal microenvironment in TID extended to that of the IEC-specific compartment, the expression of various IEC-specific soluble mediators associated with immunoregulation and inflammation and were assessed in duodenal tissues from donors with or without T1D (Table 1).

**Figure 1:**
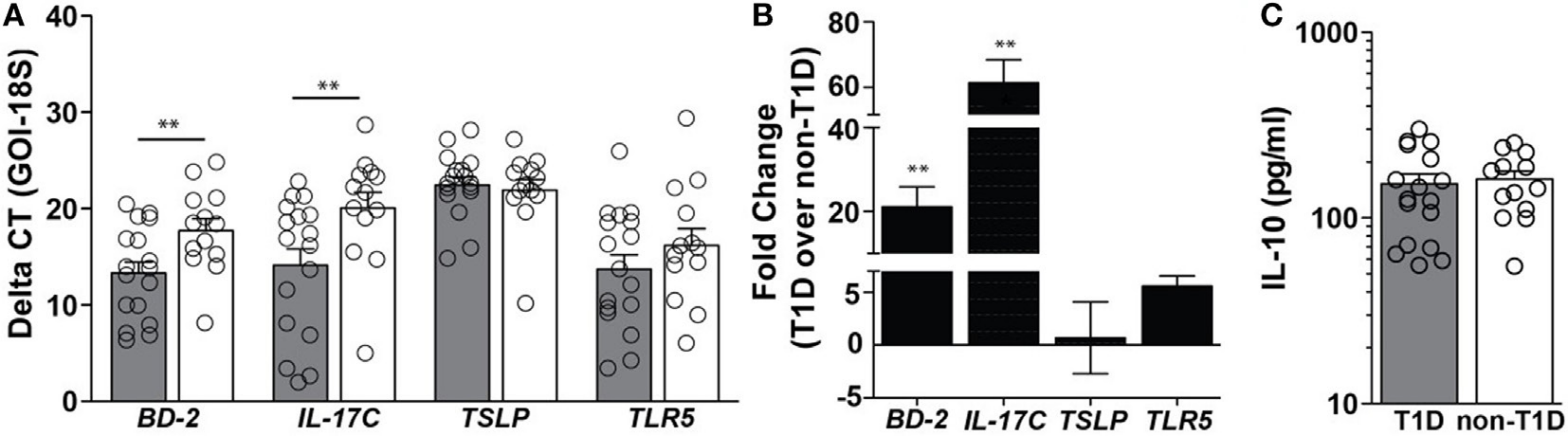
**Type 1 diabetes (T1D) is associated with elevated levels of beta-defensin 2 and IL-17C within duodenal lysates**. **(A)** Delta CT value [gene of interest (GOI)—18S] of each gene evaluated in duodenal lysates from individuals with and without T1D. Please note the higher value indicates lower expression of the GOI. **(B)** Fold change of mRNA expression of indicated genes in duodenal lysates from individuals with T1D over mRNA expression in duodenal lysates from individuals without T1D. **(C)** Protein expression of interleukin 10. Gray bars: T1D (*n* = 17); white bars: non-T1D (*n* = 13). **p ≤ 0.01.

In duodenal tissues derived from individuals with T1D, a 21-fold higher level of mRNA expression of the antimicrobial peptide beta-defensin 2 (BD-2) and a 61-fold higher level of mRNA expression of IL-17C were observed (Figures 1A,B). Importantly, these mediators were significantly elevated in the absence of similar mRNA elevations in the regulatory cytokines thymic stromal lymphopoietin (TSLP) and protein expression of IL-10 (Figures 1A–C). As BD-2 and IL-17C are known to be TLR5-response genes (21, 22), expression levels of TLR5 were also evaluated, whereby no significant differences in the mRNA levels of TLR5 were observed (Figures 1A,B).

**Figure 2:**
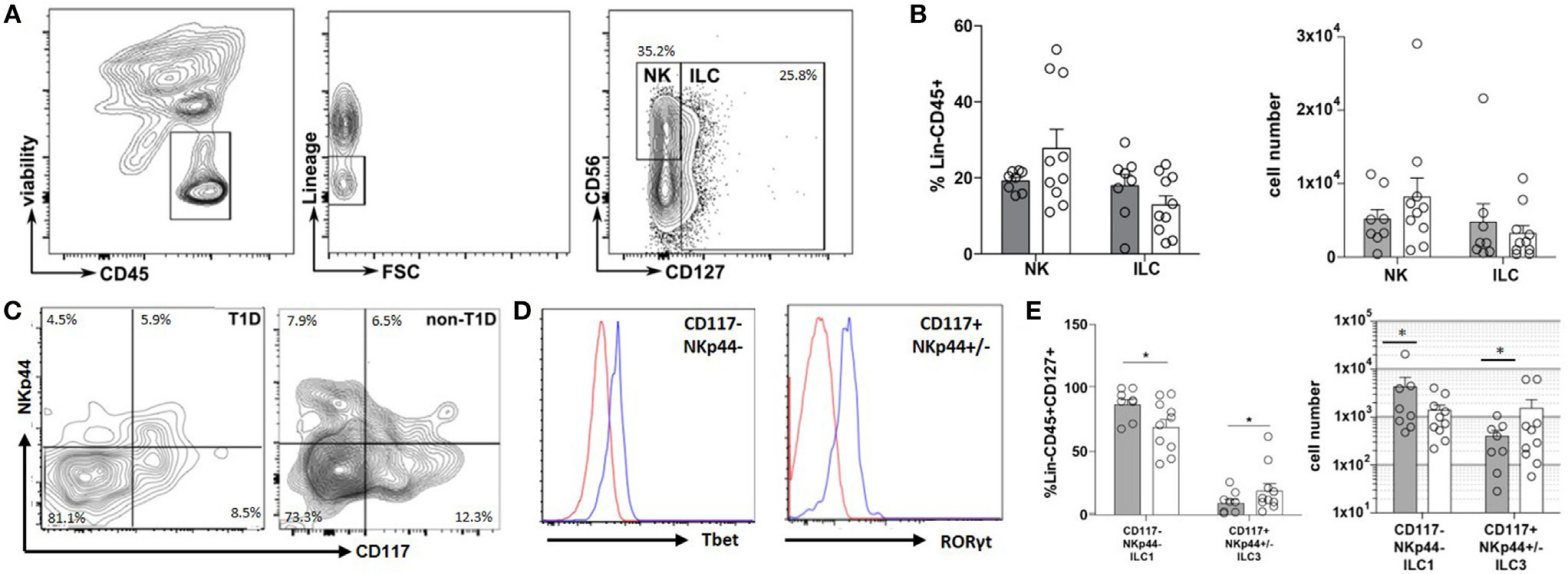
**Type 1 diabetes (T1D) is associated with duodenal accumulation of type 1 innate lymphoid cells (ILC1)**. **(A)** Intestinal ILCs gating strategy. **(B)** Frequency of and total cell numbers for intestinal NK cells (lineage negative, CD56+, CD127−) and ILC (lineage negative, CD127+). **(C)** Intestinal ILCs subset gating strategy (CD117−NKp44− Tbet+; CD117+NKp44+/−, RORγT+) **(D)** Tbet—blue histogram (Tbet high cells) is gated on CD117−NKp44−; red histogram (Tbet low cells) is gated on CD117+NKp44+/−; RORγT—blue histogram (RORγT high cells) is gated CD117+NKp44+/−; red histogram (RORγT low cells) is gated on CD117−NKp44−. **(E)** Frequency of and total cell numbers for intestinal ILC1 (CD127+CD117−NKp44−Tbet+) and ILC3 (CD117+NKp44+/−RORγT+). Gray bars: T1D (*n* = 8); white bars: non-T1D (*n* = 10). *p ≤ 0.05.

The inflammatory state of the intestinal tract is heavily regulated by and has a direct effect on the recently described ILC lineage (9). Thus, the frequency and phenotype of ILC within the duodenal tissues were also evaluated. Although no significant differences were observed in the frequency of total NK or ILC populations (Figures 2A,B), a significantly higher frequency of CD127+NKp44−CD117− cells (ILC1) concomitant with a lower frequency of CD127+CD117+NKp44+/− cells (ILC3) was observed in the T1D cohort compared to that of the non-T1D cohort (Figures 2C–E).

### T1D-Associated Intestinal Adaptive Immune Activation

**Figure 3:**
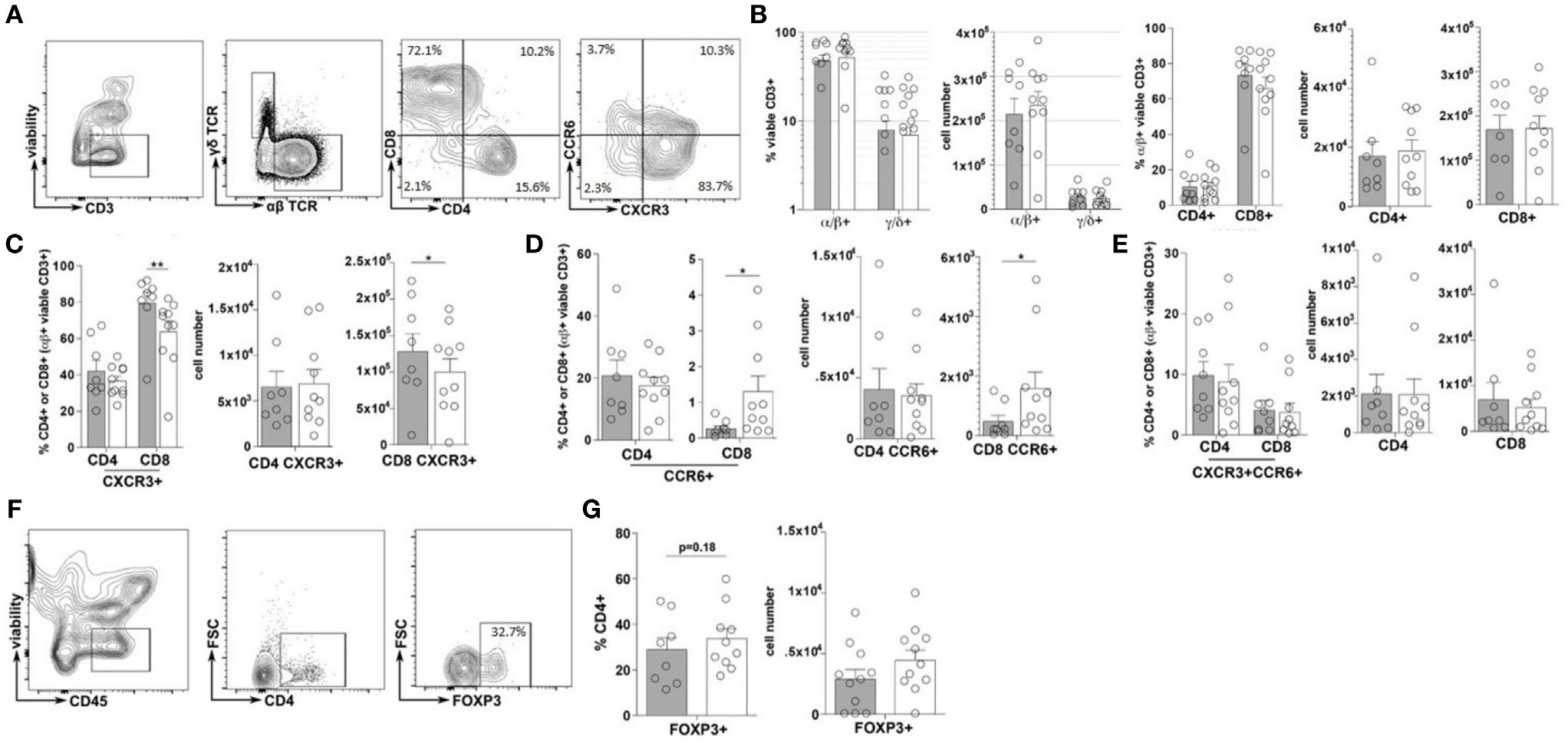
**Type 1 diabetes (T1D) is associated with elevated inflammatory duodenal CD8+ effector T-cell populations**. **(A)** Intestinal T lymphocyte gating strategy. **(B)** Frequency of and total cell numbers for CD3+ T-cells, α/β or γ/δ T-cells, and CD4+ or CD8+ T-cells. **(C–E)** Frequencies of and total cell numbers for **(C)** CD4+ or CD8+CXCR3+, **(D)** CD4+ or CD8+CCR6+, and **(E)** CD4+ or CD8+CXCR3+CCR6+ T-cells. **(F)** CD4+FOXP3+ T-cell gating strategies. **(G)** Frequency of and total cell numbers for CD4+FOXP3+ Tregs. Gray bars: T1D (*n* = 8); white bars: non-T1D (*n* = 10). *p ≤ 0.05, **p ≤ 0.01.

Immune cell plasticity within the adaptive immune cell compartments is also heavily regulated by the inflammatory state of the intestinal tract, thus the frequency and phenotype of T helper cells (CD4+) and cytotoxic T-cells (CD8+), within the duodenal tissues, were also evaluated (23). Similar to that of the total ILC populations, no significant difference in the frequencies of total CD3+, αβ+ nor γδ+ T-cells were observed between the TID and non-TID cohorts (Figures 3A–C). Furthermore, within the αβ+ T-cell populations, similar frequencies of CD4+ and CD8+ T-cells were observed (Figures 3A,B). Surprisingly, no significant differences in the frequency of duodenal CD4+CXCR3+ (Th1), CD4+CCR6+ (Th17) or CD4+CXCR3+CCR6+ (Th1/Th17), or CD4+FOXP3+ T-cells (Tregs) was observed (Figures 3A,C,E–G). Conversely, mirroring the observed phenotypic skewing of ILCs, the T1D cohort presented with a significantly higher frequency of duodenal CD8+CXCR3+ (Tc1) T-cells as well as a significantly lower frequency of CD8+CCR6+ (Tc17) T-cells (Figures 3A,D), although the frequency of CD8+CXCR3+CCR6+ (Tc1/Tc17) was similar between the cohorts (Figures 3A,E) (24, 25).

### T1D-Associated IECs Mediate Polarization of Adaptive Immunity

**Figure 4:**
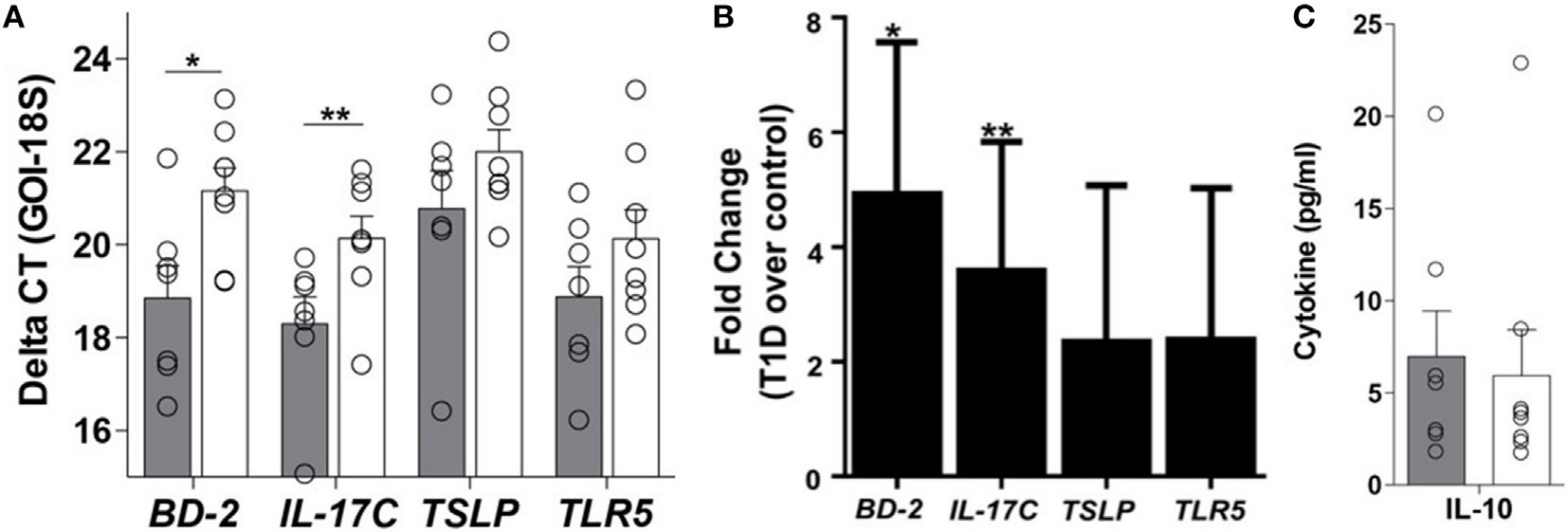
**Innate immune dysregulation is a feature of the type 1 diabetes (T1D)-derived intestinal epithelial cell (IEC)**. **(A)** Delta CT value [gene of interest (GOI)—18S] of each gene evaluated in unstimulated IEC cultures from individuals with and without T1D. Please note a higher value indicates lower expression of the GOI. **(B)** Fold change of mRNA expression of indicated genes in unstimulated IEC cultures from individuals with T1D over mRNA expression in unstimulated IEC cultures from individuals without T1D. **(C)** Unstimulated protein expression of interleukin 10. **(A,B)** Gray bars: T1D (*n* = 8); white bars: non-T1D cohort (*n* = 10).

Appropriate communication of the intestinal tract with the environment is required for local and systemic immunologic homeostasis, which is in part regulated by the IEC through its physical and biochemical interactions with innate and adaptive immune populations (13). Thus, to determine if the phenomenon observed at the whole tissue level could be a result of altered IEC-innate immune function, primary IEC cultures were established from the crypts of duodenal tissues using our previously published method (15) (Table S1 in Supplementary Material). Here, the soluble mediator gene expression profile of resting T1D-derived IEC cultures was similar to that observed in the whole tissue, whereby 5- and 3.6-fold higher mRNA levels of BD-2 and IL-17C, respectively, were observed in T1D-derived cultures, when compared to non-TID derived cultures (Figures 4A,B), without similar upregulation in immunoregulatory mediators such as TSLP or IL-10 (Figures 4A–C). Again, this altered soluble mediator expression was in the absence of significant differences in the mRNA levels of TLR5 (Figures 4A,B).

**Figure 5:**
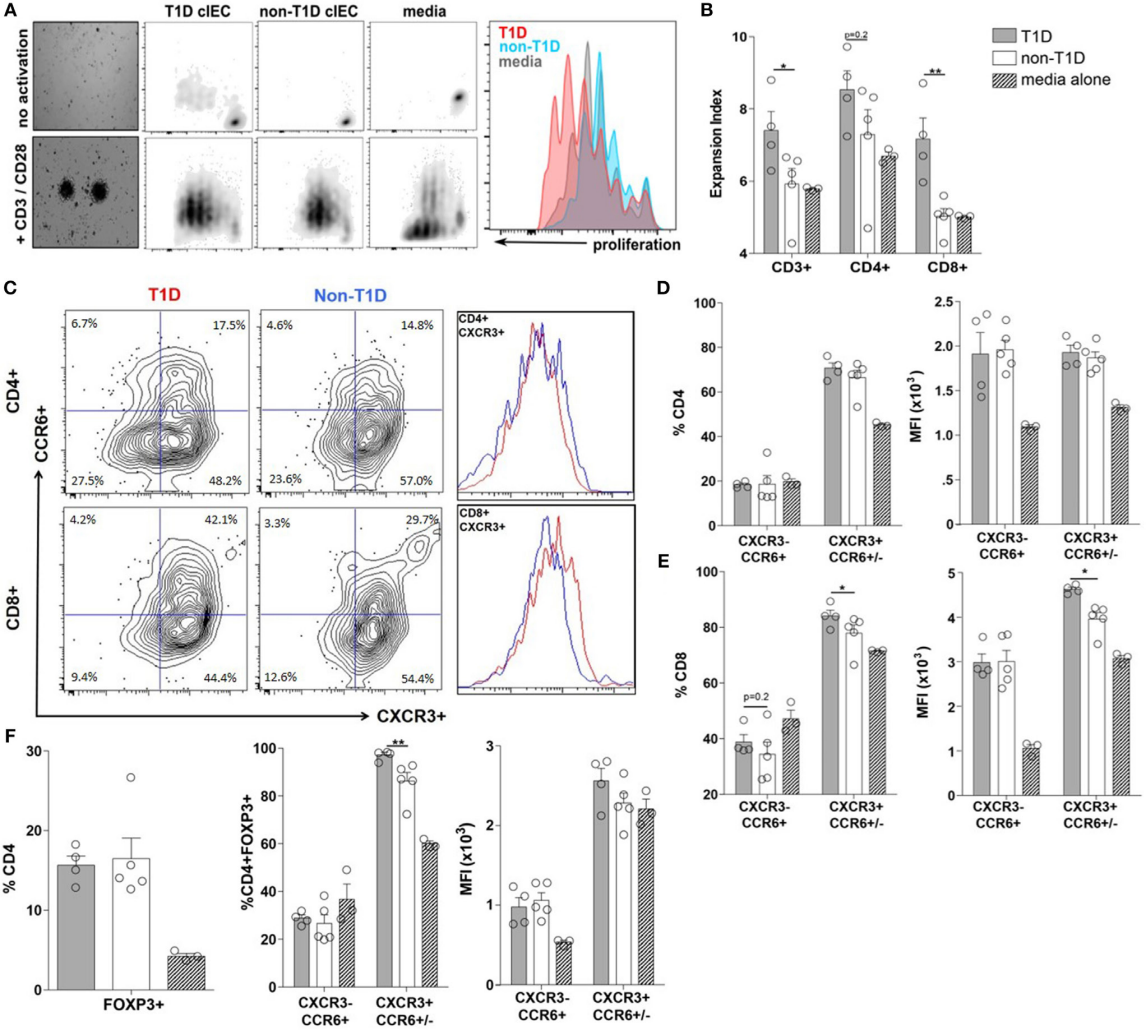
**Type 1 diabetes (T1D)-derived intestinal epithelial cell (IEC) conditioned media induce inflammatory CD8+ effector T-cell proliferation and polarization**. **(A)** Representative peripheral blood mononuclear cells proliferation data gated on CD3+ T cells following coculture with T1D- and non-T1D-derived IEC culture supernatants. **(B)** CD3+, CD4+, and CD8+ expansion index. **(C)** Representative CD4+ and CD8+ T cell gating strategy and histograms for relative levels of CXCR3+ cells red: using conditioned media from T1D IEC cultures; blue: using conditioned media from non-T1D IEC cultures. **(D,E)** Frequency and expression levels of CXCR3−CCR6+ and CXCR3+CCR6+/− among **(D)** CD4+ and **(E)** CD8+ T-cell populations. **(E)** Frequency of FOXP3+ Tregs. **(F)** Frequency and expression levels of CXCR3−CCR6+ and CXCR3+CCR6+/− among FOXP3+ Tregs. Gray bars: conditioned media from T1D IEC cultures (*n* = 8); white bars: conditioned media from non-T1D IEC cultures (*n* = 10); hatched bars: media alone (*n* = 10). *p ≤ 0.05, **p ≤ 0.01.

To determine if the T1D-associated IEC-soluble mediator milieu could directly contribute to expansion and/or polarization of the T-cell compartment observed at the whole tissue level, the proliferative capacity and polarization of PBMC from a single healthy donor was assessed following exposure to IEC-conditioned supernatants. Here, the proliferation of CD3+ T-cells was significantly enhanced when exposed to conditioned supernatants from T1D-derived IEC cultures, mostly as a result of CD8+ T-cell expansion (Figures 5A,B). While conditioned supernatants from T1D-derived IEC cultures induced a similar polarization profile of CD4+ T-cell subsets, as did conditioned supernatants from non-T1D-derived IEC cultures (Figure 5D), there was a statistically significant difference in the effects on the CD8+ T-cell populations (Figures 5C,E). Specifically, the frequency of CD8+CXCR3+CCR6+/− T-cells (Tc1) was higher following exposure to conditioned supernatants from T1D-derived cultures, whereby the expression level of CXCR3+ was also significantly elevated on a per cell basis (Figures 5C,E). Similarly, while both sources of conditioned supernatants induced an expansion of CD4+FOXP3+ T-cells (Tregs) (Figure 5F), supernatants from T1D-derived IEC cultures polarized Tregs to that of a more Th1-like phenotype as measured by a higher frequency of CD4+FOXP3+CXCR3+CCR6+/− T cells (Figure 5F).

### T1D-Associated IEC Activation Occurs in the Absence of Overt Intestinal Pathology

**Figure 6:**
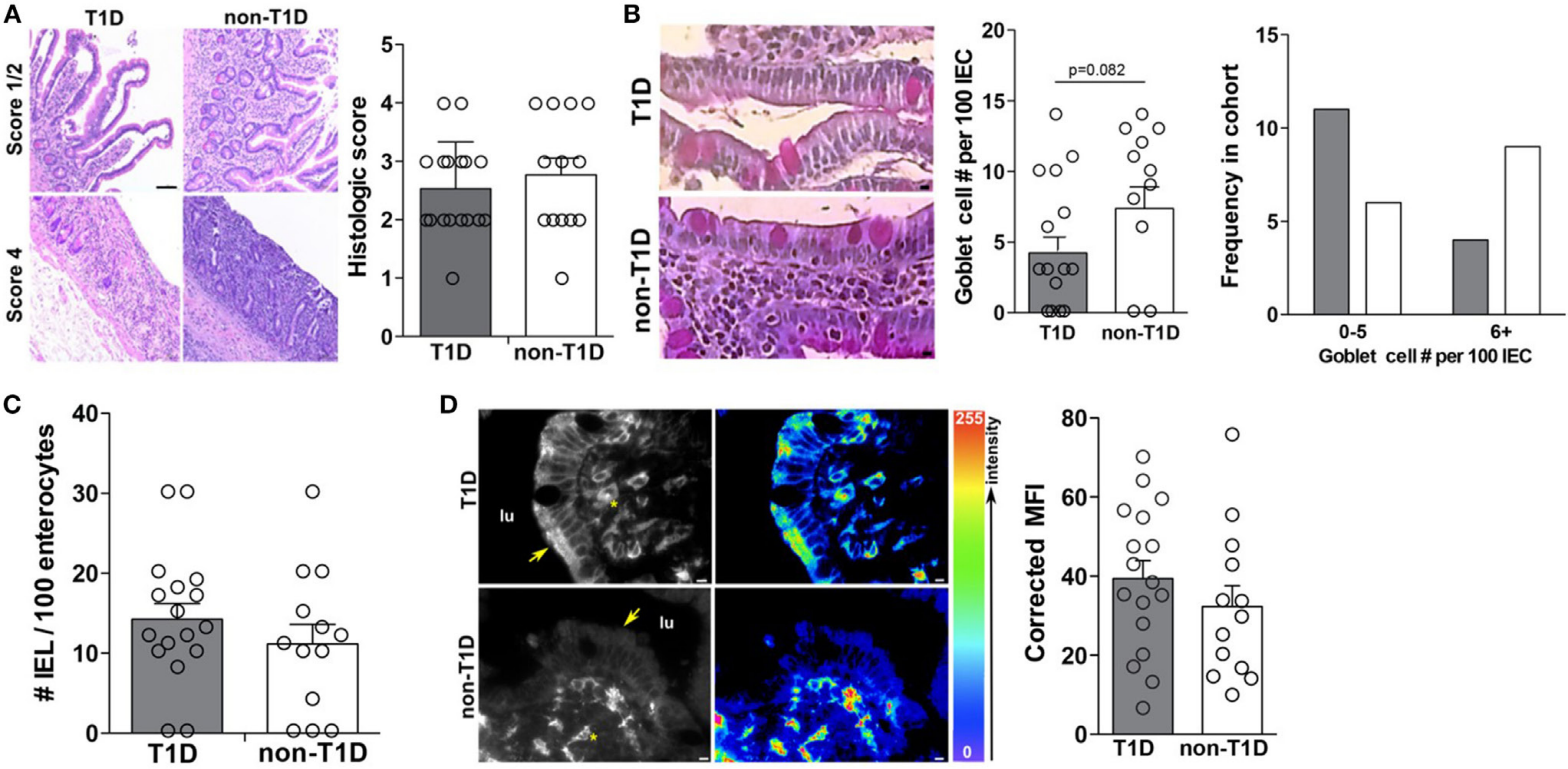
**Type 1 diabetes (T1D)-associated intestinal epithelial cell (IEC) activation occurs in the absence of overt intestinal pathology**. **(A)** Mild and severe intestinal pathology in both T1D and non-T1D cohorts. **(B)** Goblet cell frequency. **(C)** Histological analysis of intraepithelial lymphocyte frequency. **(D)** Immunofluorescence of epithelial HLA-DR expression. Arrows indicate IEC HLA expression and asterisks indicate lamina propria immune cell HLA expression. Gray bars: T1D (*n* = 17); white bars: non-T1D (*n* = 13).

As previous studies had indicated intestinal inflammation inherent in T1D cohorts, the duodenal samples of this cohort were also evaluated for overt intestinal pathology and markers of intestinal inflammation. Specifically, all tissues were evaluated for gross pathology, goblet cell and intraepithelial lymphocyte (IEL) frequency, and expression of HLA-DR. Interestingly, the absence and presence of intestinal pathology were observed in both cohorts with similar incidence (Figure 6A), whereby no statistical differences in the frequency of goblet cells (Figure 6B), IELs (Figure 6C), or HLA-DR expression (Figure 6D) was observed.

## Discussion

Non-genetic factors account for greater than 60–70% of the immunological variations between individuals, whereby a recent study demonstrated that the two major non-genetic factors contributing to immunological distance between individuals are age and cohabitation (26). These data suggest a shared environment acts in some way to bring immunoprofiles toward a convergent equilibrium (26). T1D is an autoimmune disease, whereby genetics contribute to the immunoprofiles responsible for the destruction of β-cells and loss of insulin production. Similar to those observations in health, it is long accepted that genetic susceptibility is not sufficient for T1D to manifest and only upon exposure to environmentally acquired factors is full-blown autoimmunity realized (7). More importantly, data emerging from preclinical and immunotherapy trials in T1D suggest that interfaces with the environment contribute to not only the induction of immune regulation but also to the reinforcement of pathological immune responses, although the associated mechanisms have yet to be elucidated (27, 28).

The largest environmental interface in the human body is the GI tract, a highly regulated immunological organ, which is in part responsible for the maintenance of peripheral tolerance under conditions of health. The sentinel cell of the GI tract responsible for environmental sensing is the IEC, whereby IECs have the capacity to regulate innate and adaptive immune populations (13). In addition, a number of studies have documented the contribution of immunity within GI tract to the expression of autoimmunity at distal sites, but it is unclear how the responses observed translate to antigen-specific immunity in the target tissue (29–31). Prior studies using animal models of disease and data generated from human intestinal biopsies have suggested that the intestine may either be involved in the pathogenesis of T1D or at a minimum be affected by the pathogenesis of disease (7, 8). Here, we have comprehensively characterized the duodenal IEC-specific immunological microenvironment in the human condition of T1D. Data presented here support the findings that innate and adaptive immune microenvironment of the T1D-GI tract is indeed altered at least *following* the onset of clinical disease. Importantly, our data suggest that this altered immune microenvironment is not solely due to histopathological changes of the GI tract, but more likely is at least partially dependent on the function of the IEC. In addition, within the T1D cohort, there was no correlation between sex, age, and/or duration of disease with any of the immunological parameters evaluated (data not show), again suggesting that the phenomenon observed may be a significant component of the etiology of disease. While it is clear that the soluble mediator profile of the IEC is directly contributing to the immune microenvironment at the whole tissue level and the expansion and polarization of T cell populations, we have not evaluated the totality of the soluble mediator environment at the whole tissue or IEC-specific level. For instance, retinoic acid-dependent production of TSLP and IL-22 in γδ T-cells, ILC3, and non-immune cells has been implicated in the maintenance of mucosal homeostasis, and we have yet to evaluate whether this axis is disrupted in T1D (32, 33). Studies to more fully elucidate the soluble mediator environment under conditions of health as well as T1D will be critical to the design and implementation of adjunctive therapies needed to induce proper mucosal tolerance.

The ideal therapy for T1D is one which would restore immune balance in a safe and lasting fashion (28). Both antigen-specific and non-specific tolerance-inducing therapies for T1D have been evaluated in preclinical models, which have not been successful in translating to the clinic due to the fact they were unable to confirm preclinical observations (27, 28). The reasons for this include (but are not limited to) differences in murine and human immune responses as well as lack of access to tissue compartments where important immune response may be occurring in patients (27, 34, 35). Indeed, a recent study using humanized mouse models indicated that anti-CD3 therapeutic efficacy is through a novel mechanism where human GI-tropic T-cells leave the circulation and secondary lymph organs and migrate to the small intestine and become producers of IL-10 (27). Most importantly, blockade of T-cell migration to the small intestine abolishes the treatment effects (27). These data highlight the potential role of the GI tract in secondary priming of effector T-cells, the small intestine as a potential reservoir of autoreactive T-cells, and/or differences in mechanisms of mucosal tolerance in the human condition. Intestinal inflammation can contribute to a loss of tolerance through changes in ILC, DC, and T-cell phenotypes and functions (10, 36–38). With the emergence of better technical approaches to track the fate of defined immune cell populations it has become apparent that there is substantial plasticity in ILC, T effector, and Treg subsets (39–41), whereby this plasticity is observed in both mice and humans (42). Indeed, our data demonstrate that phenotypic plasticity among GI tract-resident ILC and T-cell populations occurs in human T1D, lending to a more proinflammatory microenvironment with the T1D-duodenum. In addition, data presented here demonstrate the capacity of IEC to not only expand effector T cell populations but also to shape their phenotype and thus function. Importantly, we demonstrate that IECs from individuals with T1D preferentially expand and polarize T cells with pathogenic potential, and finally, that this can occur in the human condition. Future studies will address the effect of T1D-associated altered IEC function on ILC or DC phenotypes and functions.

It is becoming increasingly clear that cooperation among the intestinal microbiota, the intestinal barrier, and the mucosal immune system are pivotal to mucosal health and are often perturbed in T1D (43). Indeed, previous studies have demonstrated that alterations in the commensal microbiota are associated with human disease, and modulation of the microbiota can modulate disease incidence in animal models (44). As such, one limitation of our study is that we did not evaluate the microbiota associated with these duodenal tissues and thus cannot eliminate the contribution of its composition on the soluble and cellular immunoprofiles observed. However, though the microbiome directly and indirectly affects mucosal homeostasis, it is still unclear as to whether changes in the microbiome precede immunological alterations and loss of mucosal tolerance or are rather a reflection of an altered mucosal environment. Our data would suggest that altered innate sensing of IECs in T1D could have an impact on the composition of the microbiota; as such, understanding microbiota–IEC interactions in T1D represents an area of future study. An additional limitation of our study is that all data were gathered using tissues from cadaveric donors and thus represent data from different time points following diagnosis and clinical onset of disease. Therefore, our data are unable to address whether the phenomena described arise as a cause or consequence of disease. With that said, these data can be used to better inform the design, implementation, and evaluation of interventions, which are most commonly administered at these later stages of disease. Specifically, our data lend some insight into why vaccinations and attempts at establishment of mucosal tolerance in human T1D trials have been largely unsuccessful; specifically, an underlying defect in the ability to establish tolerance due to either robust mucosal inflammation and/or altered IEC-innate immune function (45).

In summary, these studies demonstrate that the perturbed innate immune function of IECs, which serve as a first line of communication with the environment, might foster a permissive environment under which autoreactive T-cells can be expanded and polarized. Thus, we propose that while IEC-innate immune dysfunction may be an early initiating event of pathogenesis, sustained IEC-driven intestinal immune dysfunction may also explain the failure of therapies aimed at establishing oral tolerance to β-cell antigens. Addressing or correcting these alterations in intestinal innate immune function may create an intestinal immune environment conducive to therapeutic intervention and thus aid efforts to establish oral tolerance.

## Author Contributions

CG and SW conceived and designed experiments. CG, JL, ML, and MS performed the experiments. CG, JL, and MW analyzed the data and performed statistical analysis. MW and SG provided intellectual expertise in innate immunity and gastrointestinal biology, respectively. CG and SW interpreted the data and drafted the manuscript. All authors reviewed and edited the manuscript. SW is the guarantor of this work and, as such, had full access to all the data in the study and takes responsibility for the integrity of the data and the accuracy of the data analysis.

## Conflict of Interest Statement

The authors declare that the research was conducted in the absence of any commercial or financial relationships that could be construed as a potential conflict of interest.
