# Supplementary material for: Intestinal Epithelial Cell Regulation of Adaptive Immune Dysfunction in Human Type 1 Diabetes
Source: Front Immunol. 2017 Jan 10;7:679. doi: 10.3389/fimmu.2016.00679 (PMC5222791; doi:10.3389/fimmu.2016.00679)
Supplement: Supplementary file 1 [file Table_1.DOCX]

# Supplementary Table 1. List of nPOD cases used in this study

| **nPOD Case** | **Status** | **Gender** | **Age** | **Duration of Disease** | **Cause of Death** | **Whole Tissue** | **Flow** | | | **IEC Culture** | **Cond. Sup.** |
| --- | --- | --- | --- | --- | --- | --- | --- | --- | --- | --- | --- |
|  |  |  |  |  |  |  | **T-Cells** | | **ILCs** |  |  |
| 6261 | T1D | M | 16 | 14 | head trauma | X | |  |  |  |  |
| 6263 | T1D | M | 34 | 21 | cerebrovascular/stroke | X | |  |  |  |  |
| 6266 | T1D | M | 30 | 23 | anoxia | X | |  |  |  |  |
| 6281 | T1D | M | 37 | 20 | anoxia | X | |  |  |  |  |
| 6285 | T1D | F | 22 | 15 | head trauma | X | |  |  |  |  |
| 6298 | T1D | M | 29 | 16 | anoxia | X | | X | X | X | X |
| 6299 | T1D | M | 32 | 23 | anoxia | X | |  |  |  |  |
| 6302 | T1D | M | 38 | 32 | anoxia | X | | X | X | X | X |
| 6306 | T1D | M | 19 | 5 | head trauma | X | |  |  |  |  |
| 6307 | T1D | F | 45 | 10 | anoxia | X | |  |  |  |  |
| 6323 | T1D | F | 22 | 6 | anoxia | X | | X | X | X | X |
| 6324 | T1D | M | 29 | 2 | anoxia | X | | X | X | X | X |
| 6327 | T1D | M | 71 | 57 | cerebrovascular/stroke | X | | X | X | X | X |
| 6328 | T1D | M | 39 | 20 | anoxia | X | | X | X | X | X |
| 6337 | T1D | F | 20 | 5 | cerebrovascular/stroke | X | | X | X | X | X |
| 6341 | T1D | M | 26 | 15 | cerebrovascular/stroke | X | | X | X | X | X |
| 6342 | T1D | F | 14 | 2 | anoxia | X | |  |  |  |  |
| 6271 | Control | M | 17 |  | head trauma | X | |  |  |  |  |
| 6288 | Control | M | 55 |  | head trauma | X | | X | X | X | X |
| 6289 | Control | M | 19 |  | head trauma | X | |  |  |  |  |
| 6290 | Control | M | 58 |  | cerebrovascular/stroke | X | | X | X | X | X |
| 6292 | Control | M | 3 |  | cerebrovascular/stroke | X | | X | X | X | X |
| 6295 | Control | F | 47 |  | cerebrovascular/stroke | X | | X | X | X | X |
| 6316 | Control | M | 6 |  | anoxia | X | | X | X | X | X |
| 6317 | Control | M | 15 |  | head trauma | X | | X | X | X | X |
| 6333 | Control | F | 27 |  | anoxia | X | | X | X | X | X |
| 6335 | Control | M | 18 |  | head trauma | X | | X | X | X | X |
| 6336 | Control | F | 14 |  | head trauma | X | | X | X | X | X |
| 6339 | Control | M | 23 |  | head trauma | X | | X | X | X | X |
| 6340 | Control | M | 9 |  | anoxia | X | |  |  |  |  |
