# Supplementary material for: Intestinal Epithelial Cell Regulation of Adaptive Immune Dysfunction in Human Type 1 Diabetes
Source: Front Immunol. 2017 Jan 10;7:679. doi: 10.3389/fimmu.2016.00679 (PMC5222791; doi:10.3389/fimmu.2016.00679)
Supplement: Supplementary file 2 [file Table_2.DOCX]

# Supplementary Table 2. List of q-RT PCR primers used in this study

| **Gene Description** | **NCBI**  **gene symbol** | **Source** | **Catalog Number** |
| --- | --- | --- | --- |
| Beta defensin-2 (BD-2) | DEFB4A | RT^2^ Primer Assay, Qiagen | PPH11010A |
| Thymic stromal lymphopoietin | TSLP | PrimeTime® qPCR Primers, IDT | Hs.PT.58.22464960 |
| Interleukin-17C | IL17C | PrimeTime® qPCR Primers, IDT | Hs.PT.58.1093657 |
| Toll-like receptor 5 | TLR5 | RT^2^ Primer Assay, Qiagen | PPH01793E |
| 18S | RNA18S5 | PrimeTime® qPCR Primers, IDT | Hs.PT.39a.22214856.g |
