# Supplementary material for: Intestinal Epithelial Cell Regulation of Adaptive Immune Dysfunction in Human Type 1 Diabetes
Source: Front Immunol. 2017 Jan 10;7:679. doi: 10.3389/fimmu.2016.00679 (PMC5222791; doi:10.3389/fimmu.2016.00679)
Supplement: Supplementary file 3 [file Table_3.DOCX]

# Supplementary Table 3. Antibodies and panels used for flow cytometric analysis

| **Antigen** | **Fluorophore** | **Clone** | **Source** |
| --- | --- | --- | --- |
| **Effector T cell Panel** | | | |
| Anti-human CD3 | APC/H7 | Leu4 | BD Biosciences |
| Anti-human αβ TCR | PE | IP26 | BioLegend |
| Anti-human γδ TCR | PE/Cy7 | B1 | BioLegend |
| Anti-human CD4 | PE/Dazzle^Tm^ 594 | RPA-T4 | BioLegend |
| Anti-human CD8 | FITC | B9.11 | Beckman Coulter |
| Anti-human CCR6 | Brilliant Violet 421^TM^ | G034E3 | BioLegend |
| Anti-human CXCR3 | APC | G025H7 | BioLegend |
| **Regulatory T cell Panel** | | | |
| Anti-human CD45 | APC/H7 | 2D1 | BD Biosciences |
| Anti-human CD4 | PerCP | OKT4 | BioLegend |
| Anti-human CCR6 | Brilliant Violet 421^TM^ | G034E3 | BioLegend |
| Anti-human CXCR3 | APC | G025H7 | BioLegend |
| Anti-human FOXP3 | AlexaFluor® 488 | 206D | BioLegend |
| Anti-mouse/human HELIOS | PE | 22F6 | BioLegend |
| **Innate Lymphoid Cell Panel** | | | |
| Anti-human Lineage Cocktail 3 (CD3,CD14,CD19 and CD20) | FITC | MφP9, L27, SK7, SJ25C1 | BD Biosciences |
| Anti-human CD45 | PerCP | HI30 | Invitrogen |
| Anti-human CD127 | APC-eFluor®780 | eBioRDR5 | eBioscience |
| Anti-human CD117 | PE-Cyanine7 | 104D2 | eBioscience |
| Anti-human NKp44 | PE | P44-8 | BioLegend |
| Anti-human CD56 | Brilliant Violet 605^TM^ | HCD56 | Biolegend |
| **Proliferation Panel A** | | | |
| Anti-human CD3 | FITC | OKT3 | BioLegend |
| Anti-human CD4 | PerCP | OKT4 | BioLegend |
| Anti-human CD8 | APC/H7 | SK1 | BD Biosciences |
| Anti-human CCR6 | PE | G034E3 | BioLegend |
| Anti-human CXCR3 | PE/Cy7 | G025H7 | BioLegend |
| **Proliferation Panel B** |  |  |  |
| Anti-human CD3 | APC/H7 | SK7 | BD Biosciences |
| Anti-human CD4 | PerCP | OKT4 | BioLegend |
| Anti-human CCR6 | APC | G034E3 | BioLegend |
| Anti-human CXCR3 | PE/Cy7 | G025H7 | BioLegend |
| Anti-human FOXP3 | AlexaFluor® 488 | 206D | BioLegend |
| Anti-mouse/human HELIOS | PE | 22F6 | BioLegend |
