# Supplementary material for: Intestinal Epithelial Cell Regulation of Adaptive Immune Dysfunction in Human Type 1 Diabetes
Source: Front Immunol. 2017 Jan 10;7:679. doi: 10.3389/fimmu.2016.00679 (PMC5222791; doi:10.3389/fimmu.2016.00679)
Supplement: Supplementary file 4 [file Table_4.DOCX]

# Supplementary Table 4. Histopathologic parameters assessed

| **Parameter and grade:** | | **Description:** |
| --- | --- | --- |
| Villous Atrophy | |  |
|  | mild |  |
|  | marked |  |
|  | complete |  |
| Severity of chronic lymphocytic infiltrate | | |
|  | mild | inflammatory lymphocytes occupying less than 1/3 of the lamina propria |
|  | moderate | inflammatory lymphocytes occupying 2/3 of the lamina propria |
|  | severe | inflammatory lymphocytes occupying the entire lamina propria |
| Grade of duodenitis | |  |
|  | 1 | normal villous structure and mild chronic inflammatory infiltration |
|  | 2 | mild villous atrophy and mild-moderate chronic inflammatory infiltration |
|  | 3 | marked villous atrophy and moderate-severe chronic inflammatory infiltration |
|  | 4 | complete villous atrophy or villous undetectable, moderate-severe chronic  inflammatory infiltration |
